# CD38: A Target for Immunotherapeutic Approaches in Multiple Myeloma

**DOI:** 10.3389/fimmu.2018.02722

**Published:** 2018-11-28

**Authors:** Fabio Morandi, Alberto L. Horenstein, Federica Costa, Nicola Giuliani, Vito Pistoia, Fabio Malavasi

**Affiliations:** ^1^Stem Cell Laboratory and Cell Therapy Center, Istituto Giannina Gaslini, Genoa, Italy; ^2^Laboratory of Immunogenetics, Department of Medical Sciences, University of Torino, Torino, Italy; ^3^CeRMS, University of Torino, Torino, Italy; ^4^Department of Medicine and Surgery, University of Parma, Parma, Italy; ^5^Immunology Area, Pediatric Hospital Bambino Gesù, Rome, Italy

**Keywords:** CD38, multiple myeloma, immunotherapy, preclinical models, clinical trials

## Abstract

Multiple Myeloma (MM) is a hematological cancer characterized by proliferation of malignant plasma cells in the bone marrow (BM). MM represents the second most frequent hematological malignancy, accounting 1% of all cancer and 13% of hematological tumors, with ~9,000 new cases per year. Patients with monoclonal gammopathy of undetermined significance (MGUS) and asymptomatic smoldering MM (SMM) usually evolve to active MM in the presence of increased tumor burden, symptoms and organ damage. Despite the role of high dose chemotherapy in combination with autologous stem cell transplantation and the introduction of new treatments, the prognosis of MM patients is still poor, and novel therapeutic approaches have been tested in the last years, including new immunomodulatory drugs, proteasome inhibitors and monoclonal antibodies (mAbs). CD38 is a glycoprotein with ectoenzymatic functions, which is expressed on plasma cells and other lymphoid and myeloid cell populations. Since its expression is very high and uniform on myeloma cells, CD38 is a good target for novel therapeutic strategies. Among them, immunotherapy represents a promising approach. Here, we summarized recent findings regarding CD38-targeted immunotherapy of MM in pre-clinical models and clinical trials, including (i) mAbs (daratumumab and isatuximab), (ii) radioimmunotherapy, and (iii) adoptive cell therapy, using chimeric antigen receptor (CAR)-transfected T cells specific for CD38. Finally, we discussed the efficacy and possible limitations of these therapeutic approaches for MM patients.

## Multiple myeloma and CD38: background

Multiple myeloma (MM) is a neoplasm characterized by a clonal expansion of malignant plasma cells (PC) in the bone marrow (BM). MM arises from pre-malignant asymptomatic proliferation of PC, that are classified as monoclonal gammopathy of undetermined significance (MGUS) and smoldering myeloma (SMM) (1). Patients with MGUS are characterized by low levels of serum M-protein (< 3 g/dL) and monoclonal PC in BM (< 10%), whereas patients with SMM display higher levels of serum M-protein (≥3 g/dL) and/or PC in the BM (≥10%). In contrast, diagnosis of MM includes the presence of end-organ damage associated with the presence of serum M-spike and/or monoclonal PC in the BM (2, 3). Malignant transformation of PC, that are derived from post-germinal center B cells, is usually driven by multiple genetic and environmental changes. Indeed, different genetic abnormalities have been detected in MM and play a role in the pathogenesis of MM, including (i) translocation of chromosome 14 (t[14;16] and t[14;4]), (ii) MYC amplification, (iii) activation of NRAS and KRAS, (iv) mutations in FGFR3 and TP53, and (v) inactivation of cyclin-dependent kinase inhibitors CDKN2A and CDKN2C (4, 5). MM accounts 1% of all cancer, and represents the second most common hematological malignancy, with 25,000–30,000 new cases per year and an incidence of 5 cases per 100,000 (6, 7). The median age of MM patients at diagnosis ranged from 66 to 70 years, and only 37% of patients display an age below 65 years (7). The median survival of relapsed MM patients has increased from 12 months (before 2000) to 24 months after 2000, due to the availability of effective treatments (8). Modern therapies, such as immunomodulatory drugs and proteasome inhibitors, have further prolonged the 5- and 10-years survival rates of MM patients, and a doubling of the median survival time has been observed in patients diagnosed in the last decade (8). However, prognosis of relapsed MM patients is still poor, and novel therapeutic approaches are urgently needed. In this context, CD38 represents a promising therapeutic target, since its expression is high and uniform on malignant PC, whereas it is relatively low on normal lymphoid and myeloid cells and on non-hematopoietic tissues. CD38 is a 45 KDa surface glycoprotein, firstly identified as an activation marker (9): successively the molecule was reported as an adhesion molecule, able to interact with endothelial CD31 (10). These finding highlighted the possibility that CD38 may act as a receptor, notwithstanding a structural ineptitude to do so. It was shown indeed that CD38 act as an accessory component of the synapse complex (11). CD38 was then identified as an ectoenzyme involved in the metabolism of extracellular nicotinamide adenine dinucleotide (NAD^+^) and cytoplasmic nicotinamide adenine dinucleotide phosphate (NADP) (12). The results is the production of Ca^2+^-mobilizing compounds, such as cyclic adenosine diphosphate [ADP] ribose, ADP ribose (ADPR) and nicotinic acid adenine dinucleotide phosphate. CD38 enzymatic activities were shown as able to rule the NAD^+^ levels and improve the function of proteasome inhibitors (13). Further, ADPR produced by CD38 can be further metabolized by the concerted action of CD203a/PC-1 and CD73, to produce the immunosuppressive molecule adenosine (ADO). This feature points out the role of CD38 in the escape of tumor cells from the control of the immune system (14).

## CD38-targeted immunotherapeutic strategies: rationale, applications and limitations

It has been demonstrated that conventional therapies, such as vincristine and doxorubicin, induce the expression of multidrug resistance genes and p-glycoprotein in tumor cells, that become resistant to different drugs (15). Thus, conventional therapies may be combined with immunotherapeutic strategies targeting CD38 to improve their efficacy. Indeed, it has been already demonstrated that combined therapies simultaneously target multiple pathways and prevent escape/resistance mechanisms of tumor cells. Moreover, combination of tumor-specific mAbs and standard chemotherapy is already a standard-of-care in several hematologic (Hodgkin's lymphoma and CLL) and solid (breast cancer and colon carcinoma) tumors (16).

In the context of MM, we have recently demonstrated that, within the bone niche, only PCs express CD38 at high levels. Moreover, CD38 expression can be detected on monocytes and early osteoclast progenitors but not on osteoblasts and mature osteoclasts, thus suggesting that CD38 expression was lost during *in vitro* osteoclastogenesis. Accordingly, we found that Daratumumab inhibited *in vitro* osteoclastogenesis and bone resorption activity from BM total mononuclear cells of MM patients, targeting CD38 expressed on monocytes and early osteoclast progenitors (17). In addition, several studies reported that anti-CD38 mAbs are able to deplete CD38^+^ immunosuppressive cells, such as myeloid-derived suppressor cells, regulatory T cells and regulatory B cells, leading to an increased anti-tumor activity of immune effector cells (18, 19).Thus, these data provide a rationale for the use of an anti-CD38 antibody-based approach as treatment for MM patients.

However, CD38 is known to be also detectable on other normal cell subsets, such as NK cells, B cells and activated T cells and the use of anti CD38 abs could thus affect the activity of normal cells. NK cells specifically play a pivotal role for the therapeutic effects of anti-CD38 mAbs, since they mediated antibody-dependent cell-mediated cytotoxicity (ADCC) and antibody-dependent cellular phagocytosis (ADCP). This issue can be addressed by using anti-CD38 F(ab')2 fragments to protect normal cells from subsequent anti-CD38 mAb-mediated lysis, or by infusion of *ex-vivo* expanded NK cells (20).

Another possible limitation of CD38-targeted therapy may be represented by the variable expression of CD38 on malignant PC. In particular, CD38 expression may be downregulated following the first infusions of anti-CD38 mAbs, favoring immune escape and disease progression (21). On this regard, combined therapy has been proposed to increase CD38 expression on malignant cells, using a pan–histone deacetylase inhibitor (Panobinostat) (22) or all-trans reticnoic acid (ATRA) (23). These studies have demonstrated that anti-CD38 mAb-mediated ADCC dramatically increased *in vitro* after the treatment, following the up-regulation of CD38 expression on MM cells (22, 23).

Anti-CD38 treatment may also generate resistance and induce tumor immune escape, through the up-regulation of two complement inhibitor proteins, CD55 and CD59 on MM cells. However, Nijhof and coworkers have demonstrated that ATRA treatment is also able to reduce CD55 and CD59 expression on anti-CD38-resistant MM cells, thus supporting the use of a combined therapy to improve complement-mediated cytotoxicity (CDC) against malignant cells (21).

In the last years, several novel immunotherapeutic approaches have been tested for MM patients, using CD38 as target, both in preclinical models and in clinical trials. These strategies include (i) mAbs specific for CD38, (ii) radioimmunotherapy, using radionuclides targeted to CD38 molecule, and (iii) adoptive cell therapy, using T cells transfected with a chimeric antigen receptor (CAR) specific for CD38.

### Anti-CD38 mAbs

**Figure 1:**
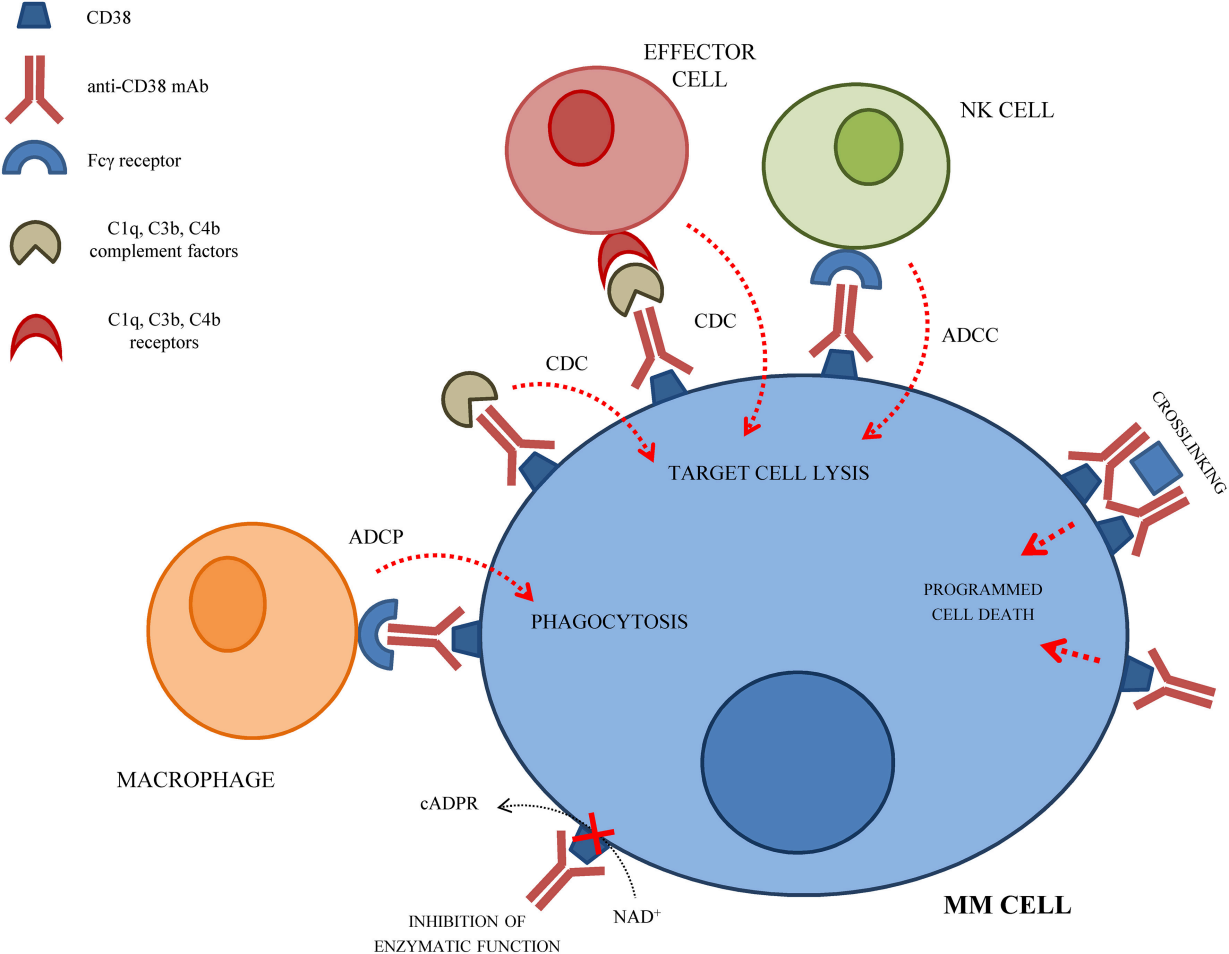
Schematic representation of the mechanism(s) of action of anti-CD38 mAbs on MM cells.

Development of mAbs against CD38 started in 1990 and anti-CD38 mAbs have been tested as immunotherapeutic strategy for MM patients, so far with limited beneficial effects. The anti-tumor effect of anti-CD38 mAbs is related to their ability to induce ADCC, CDC and ADCP of opsonized CD38^+^ cells. Moreover, anti-CD38 mAbs can induce a direct apoptosis of CD38^+^ MM cells via Fc-γ receptor-mediated crosslinking (24). Crosslinking of anti-CD38 mAbs on MM cells leads to clustering of cells, phosphatidylserine translocation, loss of mitochondrial membrane potential, and loss of membrane integrity. This effect is called homotypic aggregation, and may be related or not to caspase-3 cleavage (25). The mechanism(s) of action of anti-CD38 mAbs on MM cells are represented in Figure 1.

Here, we summarized novel findings obtained using anti-CD38 mAbs as therapeutic strategy for MM *in vitro*, in preclinical studies and, finally, in clinical trials.

#### Daratumumab

Daratumumab is a human anti-CD38 mAb, which is able to trigger ADCC and CDC *in vitro* against CD38^+^ tumor cells, using either autologous or allogeneic effector cells. Daratumumab-mediated ADCC and CDC *in vivo* is not affected by the presence of BM stromal cells, thus suggesting that this mAb can kill MM tumor cells in a tumor-preserving BM microenvironment. Moreover, Daratumumab is able to inhibit tumor growth in xenograft models at low doses (26). Another study demonstrated that Daratumumab is able to trigged programmed cell death (PCD) of MM CD38^+^ cells when cross-linked *in vitro* by secondary mAbs or via an FcγR. Moreover, in a syngeneic *in vivo* tumor model, Daratumumab is able to induce PCD of MM cells, through the cross-linking mediated by both inhibitory FcγRIIb and activating FcγRs. These data suggested that the therapeutic effect of Daratumumab may be at least in part related to the induction of PCD of MM cells through cross-linking (25). The interaction between soluble Daratumumab and FcRs appears critical for the action of the antibody. The marked polar aggregation is followed by a significant release of microvesicles (MV) (27). Generation of MV is a physiological event: the difference with the same MV after antibody treatment is the fact that they are covered with the therapeutic IgG. This makes their destination mandatory to FcR-expressing cells and tissues (28). CD38 is expressed at high levels in BM niche only by PC. However, its expression can be detected at lower levels also on monocytes and early osteoclast progenitors, but not on mature osteoblasts and osteoclasts, since CD38 expression is downregulated during *in vitro* osteoclastogenesis (17). Consistently, it has been demonstrated that Daratumumab reacts with CD38 expressed on monocytes and inhibited *in vitro* osteoclastogenesis and bone resorption activity from BM total mononuclear cells (MNC) of MM patients, by targeting CD38^+^ osteoclast progenitors. Thus, Daratumumab may be effective also to prevent osteoclastogenesis induced by MM (17). The anti-tumor efficacy of Daratumumab may be increased by the combination with immunomodulatory drugs. One study analyzed the combined effect of human anti-CD38 mAb Daratumumab and lenalidomide, a drug that is able stimulate the immune system and to induce apoptosis of tumor cells and inhibition of angiogenesis. They have demonstrated that effector cells derived from peripheral blood (PB) MNC from healthy individuals pretreated with lenalidomide displayed *in vitro* an increased ADCC mediated by Daratumumab against primary CD38^+^ MM cells and UM-9 MM cell line. Same results were obtained using BM MNC of MM patients, thus indicating that lenalidomide can increase Daratumumab-mediated lysis of MM cells by activating autologous effector cells within the natural environment of malignant cells. Finally, they have demonstrated an increased Daratumumab-dependent ADCC against MM cells using PB derived from lenalidomide-treated MM patients as effector cells. These data suggested that the combination of lenalidomide and Daratumumab may represent an effective novel therapeutic strategy for MM patients (29). This conclusion was confirmed by another study, where Daratumumab was combined with lenalidomide and bortezomib (30). Daratumumab induced lysis of (i) MM cells that were resistant to lenalidomide and bortezomib and (ii) primary MM cells using BM MNC derived from MM patients that were refractory to lenalidomide and/or bortezomib treatment. This study confirmed that lenalidomide (but not bortezomib) synergistically enhanced Daratumumab-mediated lysis of MM cells through activation of NK cells. Moreover, the combination of daratumumab with lenalidomide effectively reduced the growth of primary MM cells from a lenalidomide- and bortezomib-refractory patient *in vivo* using a xenograft model (30). We summarized the clinical results obtained with Daratumumab in a recent Review article (31).

#### Isatuximab

Isatuximab (formerly known as SAR650984) is a humanized anti-CD38 mAb that exerts a strong pro-apoptotic activity independent of cross-linking agents, and potent anti-tumor activity related to CDC, ADCC and ADCP. These functions are equivalent *in vitro* to those observed for rituximab in CD20^+^ and CD38^+^ models. Moreover, isatuximab is able to partially inhibit ADP-ribosyl cyclase activity of CD38, through an allosteric antagonism (32). Additional mechanism of action have been characterized by Jiang et al., who have demonstrated that isatuximab is able to induce homotypic aggregation-associated cell death in MM cells, that is related to the level of CD38 expression on cell surface and depends on actin cytoskeleton and membrane lipid raft (33). Isatuximab and its F(ab)'2 fragments also induce (i) apoptosis of MM cells highly expressing CD38, through the activation of caspase 3 and 7, (ii) lysosome-dependent cell death by enlarging lysosomes and increasing permeabilization of lysosomal membrane, and (ii) upregulation of reactive oxygen species. It has been also demonstrated that SAR650984-mediated killing of MM cells is enhanced by the antitumoral drug pomalidomide, even in MM cells resistant to pomalidomide/lenalidomide (33). Feng and coworkers have demonstrated that isatuximab is able to decrease the frequency of CD38^*hi*^ Treg and to increase the frequency of CD4^+^CD25^−^ T cells. Treatment with isatuximab downmodulate Foxp3 and IL10 in Tregs and restores proliferation and function of T cells. Furthermore, isatuximab increases MM cell lysis by CD8^+^ T and NK cells *in vitro* (34). MM cells are able to induce the expansion of CD38^*hi*^ Tregs *in vitro* when cultured with CD4^+^CD25^−^ T cells. In this context, isatuximab is able to inhibit the expansion of inducible Tregs by MM cells and stromal cells, by inhibiting cell-to-cell contact and release of TGFβ/IL10. Thus, this study demonstrated that isatuximab, through CD38 targeting, is able to revert MM-induced immunosuppression and to restore anti-MM immune effector cell functions (34). Finally, it has been demonstrated that isatuximab was effective to eradicate malignant cells *in vivo* in xenograft models of different hematological CD38^+^ human tumors, including MM. This anti-tumor activity was more potent than that of bortezomib in MM xenograft models set up using NCI-H929 and Molp-8 MM cell lines. More importantly, isatuximab demonstrated a potent pro-apoptotic activity against CD38^+^ human primary MM cells (32). Taken together, these findings supported the use of isatuximab in phase 1 clinical studies for MM patients, alone or in combination with other drugs such as pomalidomide or lenalidomide.

#### CD38-specific chimeric mAbs and nanobodies

In the past, several anti-CD38 mAb have been developed and tested for their ability to induce ADCC and CDC against CD38^+^ MM cells. Stevenson and coworkers have developed a chimeric anti-CD38 mAb, composed by the Fab portion of OKT10 murine mAb linked to a human IgG1 Fc fragment. This chimeric mAb, but not the parental mAb, mediated ADCC using human mononuclear effector cells, and displayed limited side effects on other CD38^+^ cell populations (i.e., NK cells and granulocyte/macrophage or erythroid progenitor cells). Chimeric mAb induced ADCC using cells isolated from 14 MM patients subjected to various chemotherapeutic regimes, and such function was similar to that observed in normal individuals, thus suggesting that treatment with anti-CD38 chimeric antibody may be effective in these patients (35). Similarly, Ellis and coworkers developed a humanized IgG1 mAb and a chimeric mAb (composed by mouse Fab cross-linked to two human gamma 1 Fc fragments) against CD38. Both mAbs efficiently directed ADCC against CD38^+^ cell lines, without down-modulating CD38 expression or enzymatic activity, thus representing a promising therapeutic strategy against MM and other diseases involving CD38^+^ cells (36).

More recently, different studies have been aimed at the generation of novel mAbs targeting CD38. In one of these studies, a series of nanobodies against CD38 with high affinities have been generated. The authors identified the epitopes that bind these nanobodies on the carboxyl domain of CD38 molecule. Next, they binded these nanobodies to fluorescent proteins to quantify CD38 expression then confirming the higher CD38 expression on MM cells as compared to normal leukocytes. More importantly, they have generated an immunotoxin, binding nanobodies with a bacterial toxin, that displayed a highly selective cytotoxicity against patient-derived MM cells and MM cell lines, even at very low concentrations. Such effect can be further enhanced by stimulating CD38 expression using retinoid acid. These results suggested that these anti-CD38 nanobodies may represent a novel diagnostic and therapeutic tool for MM patients (37). The development of anti-CD38 nanobodies has been carried out also by Fumey and coworkers. They have identified 22 nanobody families specific for CD38 molecule from llamas immunized with recombinant non-glycosylated CD38 ecto-domain, using a phage display technology (38). They performed cross-blockade analyses by flow cytometry using CD38-transfected cells, and an in-tandem epitope binding using CD38 molecule immobilized on biosensors, demonstrating that these nanobody families recognize three different non-overlapping epitopes, with four nanobody families showing a complementary binding to Daratumumab. Three nanobody families inhibit the enzymatic activity of CD38 *in vitro*, while two other families act as enhancers. All nanobodies also recognized native CD38 on tumor cells and lymphoid cells (T, B, and NK cells), and some of them still recognized tumor cells after opsonization with daratumumab, thus suggesting that these nanobodies recognized a different epitope. Finally, fluorochrome-conjugated CD38 nanobodies efficiently reach CD38^+^ tumors in a rodent model within 2 h after intravenous injection, thus allowing *in vivo* tumor imaging. This study suggested that anti-CD38 nanobodies may be effective for the modulation of CD38 enzymatic activity and for the diagnosis of CD38-expressing tumors, also in patients treated with daratumumab (38). Barabas and colleagues have developed novel anti-CD38 mAbs by injecting an immune complex, composed by CD38 antigen and homologous anti-CD38 lytic IgG mAbs, in rabbits. Recipient rabbits produced mAbs with the same specificity against CD38 antigen. Such mAbs demonstrated *in vitro* a potent agglutinating, precipitating and lytic function. Moreover, in the presence of complement, donor and recipient rabbits' immune sera lysed CD38^+^ MM cells *in vitro*. Thus, they demonstrated that this “third vaccination” method has good potential for MM therapy (39). Moreover, they have demonstrated that passive immunization of SCID mice injected subcutaneously with human MM cells with heterologous anti-CD38 IgG antibody containing serum significantly decreased cancer growth in the presence of complement, thus confirming the efficacy of this methods also in preclinical models (40).

### Radioimmunotherapy

Since malignant PC are very radiosensitive, CD38 has been used as target for radioimmunotherapy (RIT) in preclinical models of MM. Green and coworkers investigated both conventional RIT (directly radiolabeled antibody) and streptavidin-biotin pretargeted RIT (PRIT) directed against CD38 as therapeutic approach to deliver radiation doses sufficient for MM cell eradication. They demonstrated that the biodistribution was increased using PRIT as compared to conventional RIT. They achieved a tumor/blood ratio of 638:1 24 h after PRIT, whereas ratios never exceeded 1:1 with conventional RIT. (90)Yttrium absorbed dose displayed an excellent target/normal organ ratios (6:1 for kidney, lung and liver; 10:1 for whole body). Moreover, they observed an objective remission of MM in 100% of mice treated with doses ranging from 800 to 1,200 μCi of anti-CD38 pre-targeted (90)Y-DOTA-biotin 7 days after the treatment, with a complete remission at day 23, with undetectable tumor masses. Moreover, 100% of mice bearing MM xenografts treated with 800 μCi of anti-CD38 pre-targeted (90)Y-DOTA-biotin achieved a long-term tumor-free survival (more than 70 days) compared with 0% in the control group (41). Since immunogenicity and endogenous biotin blockade may limit the clinical translation of PRIT, the authors developed a new approach based on the use of an anti-CD38 bispecific fusion protein conjugated with 90Y. This protein eliminates the interference due to biotin and is less immunogenic, and demonstrated an excellent blood clearance and targeting of MM cells in xenograft models. Indeed, they demonstrated a high tumor-absorbed dose and, more importantly, a high tumor-to-normal organ dose ratios (7:1 for liver and 15:1 for lung and kidney), thus demonstrating that fusion protein targets tumor cells but not normal tissues. They obtained a 100% of complete remissions at day 12 and 80% of mice cured at optimal doses (1,200 μCi), thus demonstrating an efficacy of the fusion protein equal to streptavidin-biotin-based PRIT. Furthermore, bispecific proteins display a superior efficacy as compared to the latter method, in terms of overall survival, using lower radiation doses (600–1,000 μCi). Thus, bispecific PRIT represents an attractive candidate for clinical translation, especially for MM patients with refractory disease, which typically retained sensitivity to radiation (42). Teiluf and coworkers tested radioimmunoconjugates, consisting of the α-emitter ^213^Bi conjugated to anti-CD38 mAb in preclinical models of MM. ^213^Bi-anti-CD38 mAb was effective in the induction of DNA double-strand breaks in different MM cell lines, inducing apoptosis, cell cycle arrest and mitotic arrest, with subsequent mitotic catastrophe. The anti-tumor effect of therapeutic strategy correlated with the expression level of CD38 on MM cell lines. More importantly, they demonstrated that mice bearing MM xenografts treated with ^213^Bi-anti-CD38 mAb display a limited tumor growth via induction of apoptosis in tumor tissue, and a significantly prolonged survival compared to controls. Moreover, no signs of ^213^Bi-induced toxicity was observed in the major organ systems (43). These studies suggest that CD38-targeted RIT may represent a promising therapeutic tool for MM patients.

### Cellular therapy

Recent findings suggest that CD38 may represent a good target for antigen-specific adoptive cell therapy. Indeed, T cells expressing CAR have been successfully used in several clinical trials for solid and hematological tumors (44). Moreover, CAR T cells specific for different MM associated antigens, such as CS1 (45), B-cell maturation antigen (46), SLAMF7 (47), and CD19 (48) proved to be effective in preclinical models and/or in clinical trials. Mihara and coworkers developed anti-CD38 CAR T cells through retroviral vector-mediated transduction of the transmembrane domain of CD8α, the intracellular domains of 4–1BB and CD3ζ and anti-CD38 single-chain variable domain (scFv). Anti-CD38 CAR T cells displayed cytotoxic activity *in vitro* against either MM cell lines or primary MM cells isolated from patients. Thus, these cells may represent a powerful therapeutic tool in preclinical models of MM (49). This issue was addressed by Drent et al., who tested anti-CD38 CAR T cells *in vivo* using a xenotransplant model (using UM9 MM cell line), in which MM cells were grown in a humanized BM microenvironment. Anti-CD38 CAR T cells demonstrated a potent anti-tumor effect when administered intravenously or intratumorally, thus suggesting that these cells efficiently migrate, infiltrate, and eliminate human MM tumors growing in their natural niche. This study demonstrates that CAR mediated targeting of CD38^+^ MM cells represents a promising therapeutic strategy for MM patients (50). The same authors tested different antibody sequences, and demonstrated that anti-CD38 CART T cells are able to proliferate, to secrete pro-inflammatory cytokines and to lyse malignant cells, irrespective of the donor and antibody sequence. Moreover, they demonstrated that CAR T cells lyse the CD38^+^ fractions of CD34^+^ hematopoietic progenitor cells, monocytes, natural killer cells, and to a lesser extent T and B cells. However, they did not inhibit the outgrowth of progenitor cells into myeloid lineages and, furthermore, they were effectively controllable with a caspase-9-based suicide gene, thus guaranteeing the safety of this approach (51). In this line, the same authors recently developed anti-CD38 CAR T cells with a lower affinity for CD38 antigen. They used the “light-chain exchange” technology to combine the heavy chains of two high-affinity CD38 antibodies with 176 different germline light chains, thus generating more than 100 new antibodies with a lower affinity (10- to 1,000- fold) to CD38. Among them, they identified eight antibodies and they isolated the corresponding single-chain variable fragments to generate new anti-CD38 CAR T cells. These cells displayed a 1,000-fold reduced affinity for CD38, and were able to proliferate, produce Th1-like cytokines and, more importantly, to lyse CD38^*hi*^ MM cells but not CD38^low^ normal cells, either *in vitro* or *in vivo*. Thus, this approach allow to generate CAR T cells highly specific for tumor-associated antigens that are also expressed at low intensity by normal cells (52). These studies confirmed that anti-CD38 CAR T cells may represent a novel and effective therapeutic tool for MM patients. Indeed, three clinical trials based on CD38 CAR T cells are currently recruiting MM patients (www.clinicaltrials.gov).

A limitation on the use of CD38-specific CAR T cells may be represented by a possible toxicity of this approach, due to the presence of CD38 on normal cells, such as NK cells, activated T cells and B cells, as mentioned before. In this line, Drent et al. designed a novel class of doxycycline (DOX)-inducible CD38-specific CAR T cells, that are rapidly inactivated by low doses of DOX, allowing to control off-tumor effects within 24 h. Thus, this strategy adds a second level of safety in CAR T cell-mediated therapy of MM patients, allowing to control the activity of CAR T cells without destroying them permanently (53). Another possible limitation is represented by the variable expression of CD38 on myeloma cells. As mentioned before, ATRA may be administered in combination with CD38-specific CAR T cells to up-regulate CD38 expression on malignant cells and consequently to improve CAR T cell-mediated anti-tumor activity. In this line, Mihara et al. have demonstrated that ATRA increases the cytotoxic activity of anti-CD38 CAR T cells against (i) acute myeloid leukemia (AML) cell lines and (ii) primary AML blasts from patients (54).

On the other hand, the anti-tumor activity of CD38-specific CAR T cells may be enhanced through the combination of these cells with conventional therapies, such as checkpoint inhibitors. Indeed, it has been demonstrated that PD-1 inhibitor pembrolizumab (PEM) increased and/or prolonged detection of circulating anti-CD19 CAR T cells in acute lymphoblastic leukemia (ALL) patients. Consequently, anti-tumor activity of CAR T cells was dramatically improved in PEM-treated patients (55).

## Conclusions

The findings here reported confirmed that CD38 represents a good target for immunotherapeutic approaches for MM patients. Indeed, the efficacy of therapeutic strategies based on the use of mAbs or CAR T cells specific for CD38 has been demonstrated *in vitro* and in preclinical studies. More importantly, some of these therapeutic approaches have already been translated to the clinic, with promising results either as monotherapy or in combination with chemotherapeutic drugs. Currently, 23 clinical trials based on CD38 as target are ongoing (3 not yet recruiting, 12 recruiting, 6 active, and 2 completed, www.clinicaltrials.gov, Table 1).

**Table 1:** CD38-targeted ongoing clinical trials (www.clinicaltrials.gov).

**Study title**	**Interventions**	**Status**
Study to evaluate the safety and efficacy of anti-CD38 CAR-T in relapsed or refractory multiple myeloma patients	Biological: Anti-CD38 A2 CAR-T cells	Recruiting
Daratumumab (HuMax®-CD38) safety study in multiple myeloma	Drug: Daratumumab plus Methylprednisolone and Dexamethasone	Completed
Monoclonal antibodies for treatment of multiple myeloma. emphasis on the CD38 antibody Daratumumab	Drug: Daratumumab plus Lenalidomide and Dexamethasone	Completed
A Phase I/IIa study of human anti-CD38 antibody MOR03087 (MOR202) in relapsed/refractory multiple myeloma	Drug: MOR03087 phase 1 dose escalation plus Dexamethasone and others	Active, not recruiting
A study of JNJ-54767414 (HuMax CD38) (Anti-CD38 monoclonal antibody) in combination with backbone treatments for the treatment of patients with multiple myeloma	Drug: Daratumumab plusVelcade, Pomalidomide and others	Active, not recruiting
Phase II study of the CD38 antibody Daratumumab in patients with high-risk MGUS and low-risk smoldering multiple myeloma	Drug: Daratumumab	Recruiting
CAR-T cells therapy in relapsed/refractory multiple myeloma	Biological: Anti-CD38 CAR-T Cells	Recruiting
Isatuximab single agent study in japanese relapsed and refractory multiple myeloma patients	Drug: Isatuximab SAR650984	Active, not recruiting
SAR650984 in combination with Carfilzomib for treatment of relapsed or refractory multiple myeloma	Drug: SAR650984 plus Carfilzomib	Recruiting
Study of GBR 1342, a CD38/CD3 Bispecific antibody, in subjects with previously treated multiple myeloma	Biological: GBR 1342	Recruiting
Efficacy and safety study of Pembrolizumab (MK-3475) in combination with Daratumumab in participants with relapsed refractory multiple myeloma	Biological: Pembrolizumab plus Daratumumab	Not yet recruiting
SAR650984 (Isatuximab), Lenalidomide, and Dexamethasone IN Combination in RRMM patients	Drug: isatuximab SAR650984 plus lenalidomide and dexamethasone	Active, not recruiting
2015-12: a study exploring the use of early and late consolidation/maintenance therapy	Drug: Daratumumab plus carfilzomib, thalidomide, dexamethasone and others	Recruiting
Daratumumab in combination With ATRA	Drug: Daratumumab plus all-trans retinoic acid (ATRA)	Recruiting
Daratumumab in combination with Bortezomib and Dexamethasone in subjects with relapsed or relapsed and refractory multiple myeloma and severe renal impairment	Drug: Daratumumab plus bortezomib and dexamethasone	Recruiting
Study of Isatuximab Combined With Bortezomib + Cyclophosphamide + Dexamethasone (VCD) and Bortezomib + Lenalidomide + Dexamethasone (VRD) in newly diagnosed multiple myeloma (MM) non-eligible for transplant	Drug: Daratumumab plus lenalidomide, bortezomib, cyclophosphamide and others	Recruiting
SAR650984, Pomalidomide and Dexamethasone in combination in RRMM patients	Drug: Isatuximab SAR650984 plus pomalidomide and dexamethasone	Active, not recruiting
Daratumumab in Treating Patients With multiple myeloma	Biological: Daratumumab	Active, not recruiting
Daratumumab, Thalidomide and Dexamethasone in Relapse and/or refractory myeloma	Drug: Daratumumab plus thalidomide and dexamethasone	Not yet recruiting
Copper 64Cu-DOTA-Daratumumab positron emission tomography in diagnosing patients with relapsed multiple myeloma	Biological: Daratumumab plus imaging agent using positron emission tomography	Recruiting
Daratumumab in treating transplant-eligible participants with multiple myeloma	Drug: Daratumumab plus autologous hematopoietic stem cell transplantation	Recruiting
Daratumumab after stem cell transplant in treating patients with multiple myeloma	Drug: Daratumumab plus autologous hematopoietic stem cell transplantation and melphalan	Not yet recruiting
Multi-CAR T cell therapy in the treatment of multiple myeloma	Biological: Anti-CD38 CAR-T cells	Recruiting

Response rates for ongoing clinical trials with available clinical data are reported in Table 2. These studies confirmed that the combination of anti-CD38 mAbs with conventional therapies dramatically improved the clinical outcome of MM patients (56–59).Thus, further studies aimed at the characterization of novel combined therapies that include anti-CD38 immune effectors might be pivotal to design effective clinical strategies to increase progression-free and overall survival of MM patients.

**Table 2:** Response rates in CD38-targeted ongoing clinical trials.

**Study**	**Response rate**		
	**ORR**	**PR**	**CR**
Daratumumab Plus Lenalidomide and Dexamethasone (DRd) vs. Lenalidomide and Dexamethasone (Rd) (56)	DRd: 93% Rd: 76%	DRd: 78% Rd: 45%	DRd: 46% Rd: 20%
Daratumumab plus Pomalidomide and Dexamethasone (D,pom/dex) vs. Pomalidomide and Dexamethasone (pom/dex) (57, 58)	D, pom/dex: 60% pom/dex: 47%	D, pom/dex: 43% pom/dex: 32%	D, pom/dex: 17% pom/dex: 15%
Daratumumab Plus Bortezomib and Dexamethasone (DVd) vs. Bortezomib and Dexamethasone (Vd) (59)	DVd: 84% Vd: 63%	DVd: 62% Vd: 29%	DVd: 26% Vd: 10%

## Author contributions

FM analyzed data present in the literature and wrote the manuscript. ALH, FC, NG, FMal and VP contributed to the writing of the final version of the manuscript.

### Conflict of interest statement

The authors declare that the research was conducted in the absence of any commercial or financial relationships that could be construed as a potential conflict of interest.
